# Effects of vitamin D supplementation on pulmonary function in postmenopausal women following an aquatic exercise program

**DOI:** 10.1590/2359-3997000000211

**Published:** 2016-09-26

**Authors:** Rodrigo Nolasco, Linda D. F. Moreira, Danilo S. Bocalini, Fernanda C. A. O. Fronza, Rosangela Villa Marin, Marise Lazaretti-Castro

**Affiliations:** 1 Departamento de Medicina Faculdade de Medicina Universidade Federal de São Paulo São Paulo SP Brasil Departamento de Medicina, Disciplina de Endocrinologia, Faculdade de Medicina, Universidade Federal de São Paulo (Unifesp/EPM), São Paulo, SP, Brasil; 2 Universidade São Judas Tadeu São Paulo SP Brasil Fisiologia Translacional, Programa de Pós-Graduação em Educação Física e Ciências do Envelhecimento, Universidade São Judas Tadeu (USJT), São Paulo, SP, Brasil; 3 Escola de Educação Física e Esporte Universidade de São Paulo São Paulo SP Brasil Escola de Educação Física e Esporte da Universidade de São Paulo (EEFE/USP), São Paulo, SP, Brasil

**Keywords:** Spirometry, cirtometry, cholecalciferol, postmenopause

## Abstract

**Objective:**

This study sought to investigate the effects of vitamin D supplementation and aquatic exercise on pulmonary function in postmenopausal women.

**Materials and methods:**

This prospective and controlled study included 104 women (62 ± 6.5 years) divided into three groups: a control group lacking vitamin D and calcium supplementation which remained sedentary (CG; n = 17); a control group receiving vitamin D and calcium supplementation which remained sedentary (CDG, n = 33); and a group that completed aquatic exercises three times a week and received vitamin D and calcium supplementation (DTG, n = 54). Data before and after 6 months of the study were analyzed, including serum 25-hydroxyvitamin D (25(OH)D) and calcium concentrations, peak expiratory flow (PEF), forced vital capacity (FVC), and cirtometry.

**Results:**

We observed significant increases in 25(OH)D concentrations in CDG (52.9 ± 2.4 to 69.1 ± 2.2; nmol/L; p < 0.0001) and DTG groups (55.5 ± 3 to 71.5 ± 3 nmol/L; p < 0.0001). PEF increased by 7 ± 2% (p = 0.0080) in CDG group and 11 ± 2% (p < 0.0001) in DTG group, whereas FVC increased by 7 ± 2% (p = 0.0016) in the CDG group and 10 ± 2% (p < 0.0001) in the DTG group, whereas CG had no changes in any of these parameters. The increment value of cirtometry in DTG group (+43 ± 3%) were significantly (p < 0.0001) higher than those in CG (−4 ± 8%) and CDG (+4 ± 9%) groups.

**Conclusion:**

Our data suggest that vitamin D supplementation improves pulmonary function parameters in postmenopausal women.

## INTRODUCTION

The large increase in longevity in humans during the last century raised new issues in health care, especially the control of aging-related deterioration ( 1 ). Intense efforts have been exerted to obtain new knowledge that can lead to the progression of chronic degenerative diseases and to slow the functional impairment, including deterioration of lung function ( 2 ). Vitamin D and calcium are among the potential factors related to maintaining bone and muscle health, and they cannot be separated from osteoporosis prevention in postmenopausal women. Vitamin D produced in the skin via the action of sunlight is converted into calcitriol, a potent hormone, which is important for health. Although its most common effects are on bone metabolism and mineral homeostasis, its receptors have been identified in almost all tissues, suggesting that it might have other roles ( 3 , 4 ).

Lung function also appears to be influenced by vitamin D concentrations, with cross-sectional studies identifying associations of lower plasma concentrations of 25-hydroxyvitamin D (25(OH)D) with worse lung function parameters ( 5 , 6 ). During aging, a gradual decline in the respiratory system occurs, the related factors of which include increased lung compliance, a decreased number of alveoli, and respiratory muscle weakening, as well as rib cage deformities caused by increased thoracic kyphosis because of, among others, vertebral fractures ( 2 ).

Different regular physical activities, including water aerobics, have been used to preserve cardiorespiratory and musculoskeletal functions during aging ( 7 ). Moreover, research showed that the treatment of vitamin D deficiency in menopausal women can confer musculoskeletal benefits and reduce fall and vertebral fracture rates ( 8 , 9 ). In addition, aquatic exercise studies have also identified beneficial effects on pulmonary function in elderly women ( 10 , 11 ).

While conducting a protocol to test the effects of a differentiated water aerobics program (HydrOS), an opportunity arose to investigate the effects of vitamin D supplementation alone or in combination with high-intensity aquatic exercises on pulmonary function in postmenopausal women.

## MATERIAL AND METHODS

### Subjects

This was a post hoc analysis of a protocol designed to evaluate the effects of a high-intensity water aerobics program (HydrOS) in postmenopausal women ( 12 ). The initial study population consisted of women living in the city of Barueri, São Paulo who were previously sedentary and attending a service center for the elderly ( *Parque da Maturidade de Barueri* ) and who were selected from volunteers willing to participate in the study according to the following inclusion criteria: postmenopausal for ≥ 5 years, non-institutionalized, and able to understand and respond to commands during the physical and cognitive tests. The exclusion criteria were: any physical conditions that might affect their performance in the physical tests (hypothyroidism, primary hyperparathyroidism, severe osteoarthritis, rheumatic arthritis, edema or ulcer in the lower limbs); chronic kidney disease (serum creatinine > 1.4 mg/dL); diseases that could interfere with bone quality or neuromuscular functions; recent history of hip fracture (< 2 years); thyroid-stimulating hormone level < 0.05 or > 5.5 μIU/mL; dependence on alcohol, tobacco, or illicit drugs; currently receiving treatment with steroids, bisphosphonates, calcitonin, or vitamin D and its metabolites; use of estrogen or selective estrogen receptor modulators; use of drugs that could interfere with postural stability or vitamin D metabolism; uncontrolled high blood pressure; use of a prosthesis; regular physical activity in the last 6 months; serious heart disease; or complications during exercise stress testing that contraindicated physical activity.

The research protocol, as well as the informed consent form, was duly approved by the Ethics Committee of Unifesp (No. 1771/2008). All volunteers who agreed to participate in the study provided written consent.

### Design

**Figure 1:**
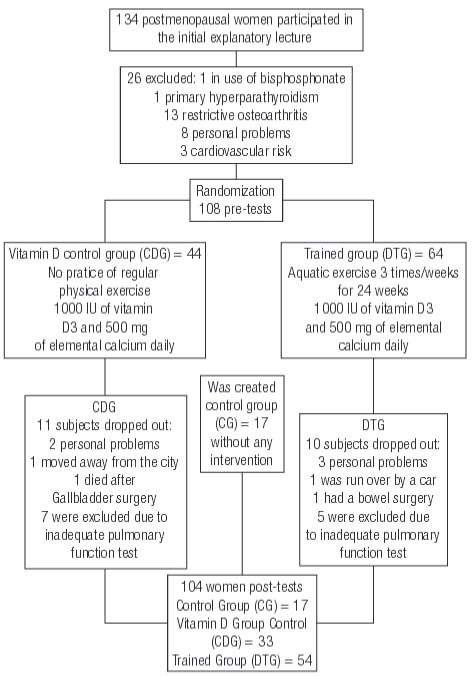
Flow of participants throughout the study.

In this prospective and controlled trial the preselection resulted in a group of 134 previously sedentary women. Of them, 26 were excluded on the basis of the described criteria ( Figure 1 ). The 108 remaining participants (58.8 ± 6.4 years) were randomly divided into two groups. The vitamin D control group (CDG, n = 33) was maintained without regular physical training, and the trained group (DTG, n = 54) participated in the HydrOS protocol over 6 months During the study period, both groups received supplementation with 1000 IU of vitamin D3 and 500 mg of elemental calcium daily. According to the same inclusion and exclusion criteria, a control group of 17 age-matched women with similar clinical characteristics was created. This group did not receive vitamin D nor participate in any physical activity program during the same period. The study design and the flow of subjects can be seen in Figure 1 .

The post hoc analysis included only patients who completed the study and who met the acceptance criteria of the American Thoracic Society for the analysis of pulmonary function tests (CG, n = 17; CDG, n = 33; and DTG, n = 54).

### Supplementation

Prior to the start of the protocol, calcium intake in all groups was evaluated by a nutritionist through 24-h diet recall ( 13 ) and the daily calcium intake was similar among the groups. None of the participants received more than 600 mg of calcium daily.

The volunteers in the control group were told to not change their lifestyle or consume any vitamin supplement during the observation period. Under medical supervision, the CDG and DTG groups received one tablet daily containing 1000 IU of cholecalciferol (vitamin D) and 500 mg of elemental calcium (as calcium carbonate) after breakfast. The participants were instructed to maintain their usual diet. During the intervention period with supplementation, the protocol adherence rate was 94% (SD ±3).

### Parameters analyzed

The selected subjects were evaluated via anamnesis, functional capacity tests, spirometry, fasting blood collection, and bone densitometry tests to evaluate body composition. Those who did not undergo all procedures and measures or attend at least 85% of the planned sessions were excluded from the study.

#### Total calcium and 25(OH)D

All volunteers underwent fasting blood collection. A radioimmunoassay method (DiaSorin, Stillwater, MN, USA) was used to determine serum 25(OH)D levels. The intra- and interassay coefficients of variation (CVs) were 5.6 and 10%, respectively. An enzymatic colorimetric method (ADVIA 1650, Bayer Ltd., Tokyo, Japan) was used to measure plasma levels of total calcium (CaT), with intra- and interassay CVs of 1.30 and 1.95%, respectively.

#### Anamnesis and physical examination

Medical and family history, ethnicity, diet, alcohol intake, smoking, and current medications were searched via anamnesis. The initial physical activity level of each participant was checked using the International Physical Activity Questionnaire, short version, already duly validated ( 14 ).

#### Body composition

Total body mass (kg) and height (cm) were obtained using an analog scale accurate to 0.1 kg (Filizola model 31, Filizola, São Paulo, SP, Brazil). Body composition was examined via whole-body dual energy X-ray absorptiometry (DXA) using a densitometer with a dual emission X-ray source (Hologic Model Discovery A, Waltham, MA, USA). All procedures involving DXA were performed in the Osteometabolic Diseases Clinic of Unifesp Endocrinology Course, and the acquisition of the exams was systematically performed using the same technique and analyzed by the same examiner.

#### Pulmonary function

Pulmonary function was assessed by spirometry using a digital portable spirometer (One Flow; Clement Clarke International, England). Patients were previously instructed about the testing process. Each individual was positioned sitting while wearing a nose clip with her feet flat on the floor and head in a neutral and raised position. After the explanation, the subject immediately performed a maximal inspiration, followed by forced expiration with the tube placed on the tongue between the teeth and the lips clenched to prevent perioral leaks. At least six full spirometric maneuvers were performed until the difference between the latest maneuvers was < 5%. The best result was employed for the analyses following the American Thoracic Society guidelines and meeting the acceptability and reproducibility criteria ( 15 ). The data were automatically transferred to an Amazon PC computer using software provided by the equipment manufacturer (ONE FLOW SOFT; Clement Clarke International, England). The following variables were considered: peak expiratory flow (PEF), forced expiratory volume in the first second of the maneuver (FEV_1_), and forced vital capacity (FVC).

The chest expansion evaluation was conducted via dynamic cirtometry at the axillary level using a 1-cm-wide measuring tape in 0.5-cm increments. The test was performed with each participant in a standing position, and the tape was placed at the axillary level around her bare chest. Each participant was instructed to perform a maximum expiration and inspiration, and the difference in centimeters between the two torso circumference values was recorded.

## Exercise protocol

The exercise sessions occurred three times a week in in a covered swimming pool with a depth of 1.20–1.40 m and temperature of 29–31°C located in *Parque da Maturidade de Barueri,* São Paulo. During the lessons, we asked the participants to stand in the pool so that the water was near the xiphoid process. To control the training intensity, three heart rate sensors (RS100 BLK; Polar, Finland) were used by three different students in each session so that at the end of 6 months, all participants had already used the heart rate sensor several times. In addition, the participants were taught how to use a Borg CR-10 category scale of perceived exertion (ranging from 0 – nothing at all, to 10 – very, very hard) used along with the measurement of the heart beat rate of the participants during the sessions ( 12 ).

During the lessons, we observed that the heart rate shown on the heart rate sensors was equivalent to the grade given by the participants on the Borg CR-10 scale. At every stage of the protocol, various muscle groups (chest and back, biceps and triceps, shoulders, quadriceps and posterior thigh, hip adductors and abductors, and calf and abdominals) were trained. The workout routine was gradually changed from two to five repetition sets of the same exercise, *e.g* ., two sets of 30 s each, then moving on to three sets of 20 s each, followed by four sets of 15 s each, and ending with five sets of 10 s each. For the cardiorespiratory exercises, we used the Borg scale adapted as follows: 60% maximum heart rate (MHR), scale level 6 for 16 minutes of the session; 70% MHR, scale level 7 for 13 minutes of the session; 80% MHR, scale level 8 for 9 minutes of the session; and 90% MHR, scale level 9 for 7 minutes of the session ( 12 ). After the 6-month intervention period with a resistance aquatic exercise program, all tests were repeated in the three groups.

## Statistical analysis

For all statistical analyses, the results were considered significant at p < 0.05. The Kolmogorov-Smirnov test was used to test data normality. The other normally distributed parameters were compared in two manners: intragroup before and after the study using Student’s *t* -test for matched samples; and intergroup before and after the study using Student’s *t* -test for independent samples. The chi-square test was used to verify the distribution of the different races of the 3 groups. One-way analysis of variance and Tukey’s post hoc test were used when necessary to identify statistical differences.

## RESULTS

Table 1 shows that the three groups were similar at baseline in terms of biometric characteristics, total calcium, and body composition. The racial distribution (white and no white) did not differ between groups (p = 0.056).

**Table 1:** Clinical features and anthropometric characteristics of the three groups at baseline and study completion

Variable	Control Group (n = 17)			Vitamin D Group (n = 33)			Trained Group (n = 54)		

	Before	After	%	Before	After	%	Before	After	%
Age	59.9 ± 2			58 ± 0.8			59 ± 6		
Weight (kg)	70 ± 3	70 ± 3	0.2 ± 0.4	74 ± 2	75 ± 3	1.3 ± 0.6	73 ± 2	73 ± 2	0.7 ± 0.4
Height (cm)	1.59 ± 2	1.59 ± 2	- 0.01 ± 0.1	1.56 ± 1	1.56 ± 1	0.12 ± 1	1.56 ± 0.1	1.56 ± 0.1	0.22 ± 0.6
BMI (kg/m^2^)	27 ± 0.1	27 ± 0.1	0.23 ± 0.40	30 ± 0.7	30 ± 0.8	0.87 ± 0.51	29 ± 0.7	29 ± 0.7	-0.48 ± 0.78
Fat mass (kg)	26 ± 1.7	26 ± 1.8	-7.0 ± 6	29 ± 1.6	29 ± 1.7	-1.3 ± 1.3	28 ± 1.1	28 ± 1.2	-1.0 ± 2
Lean mass (kg)	42 ± 17	42 ± 16	-0.5 ± 1	42 ± 16	44 ± 12	4 ± 3	43 ± 10	43 ± 10	0.8 ± 1
Fat%	37 ± 1.3	36 ± 1.5	- 1 ± 1	38 ± 1.0	38 ± 1.1	-1.5 ± 0.8	38 ± 0.7	38 ± 0.7	-1 ± 0.8
Cirtometry (cm)	2.05 ± 0.3	2.04 ± 0.2	-4 ± 5^a^	2.12 ± 0.14	2.45 ± 0.16	4 ± 9^a^	2.21 ± 0.13	3.93 ± 0.16*	43 ± 3^b^
Ca T (RV: 8.5-10.5 mg/dL)	9.23 ± 0.43	9.14 ± 0.28	-0,9 ± 2	9.50 ± 0.23	9.20 ± 0.31	-3,2 ± 4	9.61 ± 0.33	9.40 ± 0.39	-2,2 ± 3

Values are expressed as the mean ± SE of the biometric measurements and total serum calcium (CaT) levels of the control (CG), control with vitamin D and calcium supplementation (CDG), and training (DTG) groups before and after the intervention. * p < 0.0001, indicate differences detected using Student’s t-test versus before the intervention. Different letters indicate statistically significant differences (p < 0.05) on one-way analysis of variance (Tukey’s post hoc test) in relation to the percentage change between the groups. BMI, body mass index. RV = Reference values. * p < 0.05.

**Figure 2:**
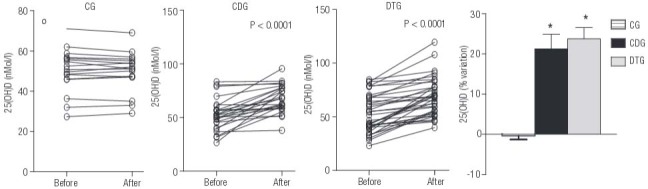
Evolution of serum 25-hydroxyvitamin D (25(OH)D) levels. Values are expressed as the mean ± SE of 25(OH)D levels in the control (CG), control with vitamin D and calcium supplementation (CDG), and training (DTG) groups before and after the intervention. * p < 0.0001, indicate differences detected using Student’s t-test versus before the intervention.

After 6 months of study, serum 25(OH)D levels similarly increased in the two groups supplemented with vitamin D3 (p < 0.001). The mean value in the CDG group increased by 21% (from 52.9 ± 2.4 to 69.1 ± 2.2; nmol/L, p < 0.0001), in the DTG group increased by 23% (from 55.5 ± 3 to 71.5 ± 3 for 3 nmol/L, p < 0.0001), and in the CG group did not change significantly (from 50.1 ± 2.6 to 49.82 ± 2.4 nmol/L, p= 0.4605) ( Figure 2 ).

**Figure 3:**
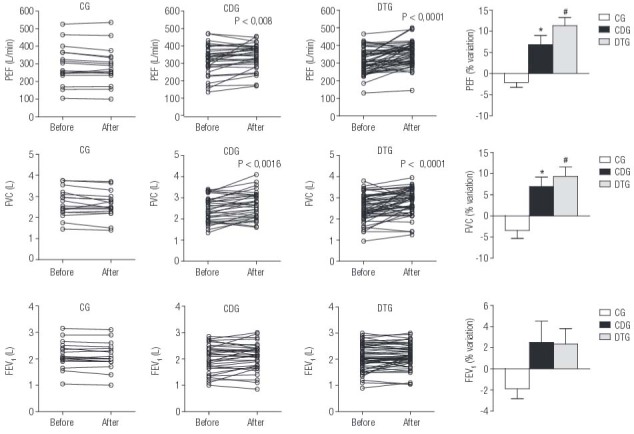
Evolution of pulmonary function test data during the study in the three groups. Values are expressed as the mean ± SE and percentage change for peak expiratory flow (PEF), forced vital capacity (FVC) and forced expiratory volume in 1 s (FEV1) in the control (CG), control with vitamin D and calcium supplementation (CDG), and training (DTG) groups before and after intervention. * p < 0.0001 and # p < 0.01, indicate differences detected using Student’s t-test versus before the intervention.

There were similarly significant improvements in PEF (L/m) and FVC (L) in both groups treated with vitamin D3 (CDG and DTG) regardless of physical training, whereas changes were not observed in the CG group ( Figure 3 ). Although FEV_1_ values were higher at the end of study than the baseline values in the vitamin D-supplemented groups, this change did not reach statistical significance ( Figure 3 ). The measure of chest expansion (dynamic cirtometry), however, revealed a significant improvement only in the group that underwent physical training ( Table 1 ).

## DISCUSSION

This study is a by-product of a broader protocol that assessed the effects of a high-intensity water aerobics program in postmenopausal women using several physical and laboratory parameters ( 12 , 16 ). Our results demonstrated that daily treatment with 1,000 IU of vitamin D3 for 6 months effectively produced a significant increase in plasma 25(OH)D concentrations in most of the volunteers. In our study, this increase on 25(OH)D levels was associated with an improvement in pulmonary function expiratory parameters (measure by spirometry), even in sedentary women, regardless of no participation in the aquatic exercise program. The trained group, additionally, was the only group to display improvement in the expandability parameter measured by cirtometry, suggesting some effect of water aerobics on this parameter ( 12 ). We have to point out that all volunteers had age-related normal values on spirometry at baseline, and both Vitamin D-treated groups improved similarly their expiratory parameters, not seen in the control group (CG). A possible explanation for the similar results on the pulmonary function parameters of DTG and CDG would be related to the increased respiratory muscle mass. The greater dynamic compression (restrictive) of the airways would lead to the fall of the flows measured at nozzle ( 17 ). The same dose of supplementation (1,000 IU/day) was used in another study ( 18 ) which was able to induce a significant increment on serum levels of 25(OH)D after 3 months (+17,6 nmol/L, in average).

The correlations between vitamin D metabolism and respiratory function have been widely studied in the literature ( 6 ). However, the results are not yet conclusive ( 19 ); rather, they remain rare in prospective intervention studies, and to our knowledge, no study has examined postmenopausal women without previous lung disease. In a randomized prospective double-blind study, supplementation with vitamin D in asthmatic children reduced the risk of exacerbations triggered by acute respiratory infection and significantly improved FEV_1_ values ( 20 ). Another prospective study in patients with chronic obstructive pulmonary disease (COPD) and vitamin D deficiency observed increases in respiratory muscle strength, reductions in the degree of dyspnea, and superior physical performance after 1 year of vitamin D supplementation ( 21 ).

Several studies observed an increased prevalence of inadequate vitamin D levels in patients with different respiratory diseases such as asthma, COPD, tuberculosis, and a number of infections of the respiratory system ( 22 - 24 ). In observational studies such as the NHANES cohort, a cross-evaluation of 14,000 adults uncovered a significant association between higher 25(OH)D levels and better results for FEV_1_ and FVC ( 5 ). Similar results were observed by other authors in 1315 Chinese children ( 25 ). A Danish longitudinal study that followed 18,507 patients of different age groups for 20 years found that lower serum 25(OH)D concentrations were associated with worse pulmonary function and an accelerated decline in FEV_1_ and FVC parameters ( 6 ).

Studies have indicated that respiratory epithelial cells express 1α-hydroxylase, which is responsible for the conversion of 25(OH)D to its biologically active form 1,25(OH)_2_D_3_, as well as the specific receptor for vitamin D (VDR) ( 26 , 27 ). Cross-sectional and prospective studies uncovered evidence associating the actions of vitamin D with increased surfactant substance levels and reduced inflammation in the respiratory tissues via VDR expressed in the airways ( 28 , 29 ). Besides, there is evidences for the action of vitamin D on the immune system, by increasing levels of LL-37 (cathelicidin), a peptide capable of promoting innate immunity and inducing the destruction of infectious agents, including *Mycobacterium tuberculosis* and influenza virus ( 19 , 29 , 30 ).

In addition, some studies linked the actions of vitamin D to inhibited proliferation of metalloproteinases of the extracellular matrix (ECM) as well as the balance of type III collagen synthesis by fibroblasts ( 31 ). The ECM of lung tissue plays a key role in the structure and mechanical properties of the lungs, thus reflecting pulmonary function ( 5 , 32 ).

Studies in rats evaluated the direct effects of vitamin D on lung growth, and they observed an increased lung volume and improved pulmonary function in these animals. These results were obtained by analyzing the forces generated by the elastic recoil of the lungs in rats with vitamin D deficiency by plethysmography and histological analysis of the lung tissue, respectively ( 28 ). Rats born to females not supplemented with vitamin D displayed structural changes, such as a significant increase in airways resistance, and changes in mesenchymal differentiation markers of the trachea compared to rats born to supplemented females ( 33 ). The direct effects of active vitamin D on lung tissue observed in these experimental studies could explain our findings in this population of women supplemented with vitamin D, even in the absence of physical training (CDG group).

The improvement observed via cirtometry only in the DTG group suggests that high-intensity aquatic exercises can increase chest expansion, probably by strengthening breathing muscles ( 17 ). This improvement may be associated with the influence of the physical properties of water, which increase voluntary breathing. The immersion of a subject in a liquid medium to the level of the xiphoid process promotes an increase of 60-65% in the work of breathing caused by increased resistance to thorax expansion produced by hydrostatic pressure and an increased blood volume in the thorax ( 34 , 35 ). This positive effect of the aquatic environment appears to favor the process of inspiratory force, as demonstrated by some studies in healthy elderly subjects ( 34 ).

Despite these findings, this project has limitations such as the discrepancy among the sample sizes, particularly the smaller number of individuals in the control group. A fourth group without vitamin D supplementation subjected exclusively to an aquatic exercise program could be used to evaluate the isolated effects of exercise on pulmonary function. There were no surveys conducted on the time of sun exposure of the study participants. However, the samplings were performed all on the same occasions, therefore, we can suppose that the potential seasonal variations of Vitamin D were similar in the 3 groups, although we have not controlled the individual quantity of exposure to sunlight. Another limiting factor was the lack of an assessment of respiratory muscle strength through measurements of inspiratory and expiratory pressures.

In conclusion, vitamin D supplementation was associated with improved spirometric parameters in postmenopausal women independent of participation in an aquatic exercise program. Aquatic exercises in turn increased chest expansion in the trained group. Based on our findings in addition to related literature, there are strong evidences that Vitamin D can play a role on pulmonary function. The increase of Vitamin D levels could also be a supporting measure for the strategies employed for preventing and treating diseases that impair pulmonary function.
